# A ferritin-based nanoparticle displaying a neutralizing epitope for foot-and-mouth disease virus (FMDV) confers partial protection in guinea pigs

**DOI:** 10.1186/s12917-024-04159-9

**Published:** 2024-07-06

**Authors:** Bingzhou Lu, Yi Ru, Rongzeng Hao, Yang Yang, Huanan Liu, Yajun Li, Yue Zhang, Yuhan Mao, Rui Yang, Yangyang Pan, Sijiu Yu, Haixue Zheng, Yan Cui

**Affiliations:** 1 College of Veterinary Medicine, Gansu Agricultural University, Lanzhou, 730070 China; 2 State Key Laboratory for Animal Disease Control and Prevention, College of Veterinary Medicine, Lanzhou University, Lanzhou Veterinary Research Institute, Chinese Academy of Agricultural Sciences, Lanzhou, 730000 China

**Keywords:** Foot-and-mouth disease virus (FMDV), Neutralizing epitope, Ferritin-based nanoparticles (FNPs), Neutralizing antibody, Protection efficacy

## Abstract

**Background:**

Foot-and-mouth disease (FMD) is a devastating disease affecting cloven-hoofed animals, that leads to significant economic losses in affected countries and regions. Currently, there is an evident inclination towards the utilization of nanoparticles as powerful platforms for innovative vaccine development. Therefore, this study developed a ferritin-based nanoparticle (FNP) vaccine that displays a neutralizing epitope of foot-and-mouth disease virus (FMDV) VP1 (aa 140–158) on the surface of FNP, and evaluated the immunogenicity and protective efficacy of these FNPs in mouse and guinea pig models to provide a strategy for developing potential FMD vaccines.

**Results:**

This study expressed the recombinant proteins Hpf, HPF-NE and HPF-T34E via an *E. coli* expression system. The results showed that the recombinant proteins Hpf, Hpf-NE and Hpf-T34E could be effectively assembled into nanoparticles. Subsequently, we evaluated the immunogenicity of the Hpf, Hpf-NE and Hpf-T34E proteins in mice, as well as the immunogenicity and protectiveness of the Hpf-T34E protein in guinea pigs. The results of the mouse experiment showed that the immune efficacy in the Hpf-T34E group was greater than the Hpf-NE group. The results from guinea pigs immunized with Hpf-T34E showed that the immune efficacy was largely consistent with the immunogenicity of the FMD inactivated vaccine (IV) and could confer partial protection against FMDV challenge in guinea pigs.

**Conclusions:**

The Hpf-T34E nanoparticles stand out as a superior choice for a subunit vaccine candidate against FMD, offering effective protection in FMDV-infected model animals. FNP-based vaccines exhibit excellent safety and immunogenicity, thus representing a promising strategy for the continued development of highly efficient and safe FMD vaccines.

## Background

Foot-and-mouth disease (FMD), caused by foot-and-mouth disease virus (FMDV), is an acute, highly contagious disease of cloven-hoofed animals. Important domesticated production animals, including cattle, pigs, sheep, goats and buffalo, plus approximately 70 species of other cloven-hoofed wildlife animals, are susceptible to FMDV [1]. FMD remains one of the most feared infectious animal diseases in countries with a highly developed livestock production industry [2]. FMDV is a nonenveloped RNA virus, belonging to the genus *Aphthovirus* within the family *Picornaviridae* [3]. It comprises seven serotypes, O, A, C, Asia-1, SAT1, SAT2, and SAT3, with multiple subtypes and variants within each serotype [4]. Vaccination has played a major role in FMD control [5]. Conventional inactivated vaccines (IV) have been widely used and have played a crucial role in epidemic control and eradication of FMDV globally [6]. Although the inactivated FMD vaccine has been very effective, there are several shortcomings, including the fact that vaccine production requires expensive high-containment biosafety level 3 facilities, it is serotype specific, the induction of cellular immunity is very limited and it is cumbersome to screen the vaccine virus strain [7, 8]. Therefore, a new approach to generate vaccines that can be produced without cultivating fully infectious viruses could provide a solution to these problems [5]. Consequently, there is a strong need to develop alternative, efficient and safe FMD vaccines. Ideally, novel vaccines should protect the host against a vast number of FMD strains, and the production process is simple and inexpensive.

To date, there is a growing body of evidence indicating that nanoparticles are desirable platforms for generating efficacious vaccines [9, 10]. Among these, self-assembling ferritin-based nanoparticles (FNPs) composed of 24 identical subunits have been highly certificated in eliciting a broadly neutralizing antibody [11–13]. *Helicobacter pylori* ferritin (Hpf) NPs are commonly used to display antigens or epitope peptides on their surface for rational vaccine design [14–16]. Due to the inherent properties of FNPs, such as being virus-sized and more readily captured by antigen-presenting dendritic cells [17, 18], the multivalent presentation of the antigen facilitates receptor clustering and subsequent activation of B cells [19, 20]. It is also a self-adjuvanting immunogen that is more effective at inducing safe and efficient humoral and cellular immune responses [19, 21]. FNPs are attractive candidate vaccine platforms for developing vaccines against several viral pathogens such as Epstein–Barr virus [22], human immunodeficiency virus [23–25], hepatitis C virus [26, 27], human respiratory syncytial virus [28], classical swine fever virus [15], Zika virus [13, 29], and SARS-CoV-2 [12, 30–32]. To our knowledge, the FMD FNP vaccine, which was generated by inserting the FMDV VP1 or G-H loop into the N terminator of Hpf and that provides partial protection in mice has been reported. The survival rate of the G-H loop-Ft group was greater than that of the VP1-Ft group [16]. The highly symmetrical and self-assembling FNP serves as a novel display and delivery platform for foreign peptide epitopes [11]. There are two sites for epitope peptide sequence insertion in Hpf: one is at the loop between Hpf helices αA and αB, and the other is at the N-terminus of Hpf [14]. However, the optimal site for epitope insertion in Hpf to achieve superior immune effects remains unclear. Therefore, identifying the most suitable location for incorporating dominant neutralizing epitopes into FNPs is of paramount importance for developing novel nanovaccines against FMDV. The primary neutralizing antigenic site in FMDV is located within the G-H loop of the VP1 capsid protein, which effectively elicits neutralizing antibodies specific to FMDV [33, 34]. The FMDV B-cell epitope VP1 (aa 140–158) has been used in dendrimer peptide B2T vaccines, elicits potent B- and T-cell-specific responses and confers partial protection in pigs against type-O FMDV challenge [35, 36]. The chimeric rabbit haemorrhagic disease virus VLP vaccine containing two FMDV-derived epitopes, a neutralizing B-cell epitope VP1 (aa 140–158) and a T-cell epitope 3 A (21–35), elicited a robust neutralizing immune response in mice and pigs and afforded partial clinical protection against FMDV challenge in pigs [37]. Importantly, these data showed that a specific and potent neutralizing antibody response was elicited by the neutralizing epitope VP1 (aa 140–158).

In this study, we evaluated the ability of FNPs as a vaccine vector for the induction of immune responses and protection against FMDV. We generated a set of chimeric FNPs by inserting FMDV-derived neutralizing epitopes individually at two different locations within Hpf. The immunogenic potential of the different FNPs was analysed in mouse and guinea pig models. Our results showed that Hpf-T34E, which displays FMDV-derived epitopes, elicited a robust neutralizing immune response in mice and guinea pigs, providing partial protection against FMDV challenge in guinea pigs. This model provides a novel strategy for subunit vaccine construction targeting FMDV dominant antigenic epitopes.

## Materials and methods

### Biosafety statement and ethics statements

All experiments related to FMDV were carried out at a biosafety laboratory-3 level (BSL-3) at the Lanzhou Veterinary Research Institute (LVRI), Chinese Academy of Agricultural Sciences (CAAS), accredited by the China National Accreditation Service for Conformity Assessment (CNAS) and approved by the Ministry of Agriculture and Rural Affairs. In the laboratory, to reduce any potential risk, the protocols were strictly followed, and all activities were monitored by professional staff at LVRI and randomly inspected by local and central governmental authorities without advance notice.

### Cells, viruses, and proteins

BHK-21 cells were retrieved from the cell line database in our lab and cultured at 37 °C in a 5% CO_2_ incubator in Dulbecco’s Modified Eagle medium (DMEM, Gibco, Waltham, MA, USA) supplemented with 10% (v/v) foetal bovine serum (FBS) (ExCell Bio, Shanghai, China) and 1% (v/v) penicillin streptomycin (pen/strep, Life Technologies). A virus stock derived from the FMDV isolate O/Mya98/BY/2010 (GenBank accession code: JN998085.1) were stored in the National Foot-and-Mouth Disease Reference Laboratory (China), which maintained the consensus sequences at the capsid protein region, was used to perform virus neutralization assays and guinea pig challenge experiments. The purified FMDV-VP1 protein was prepared in our laboratory.

### Plasmid construction

**Fig. 1:**
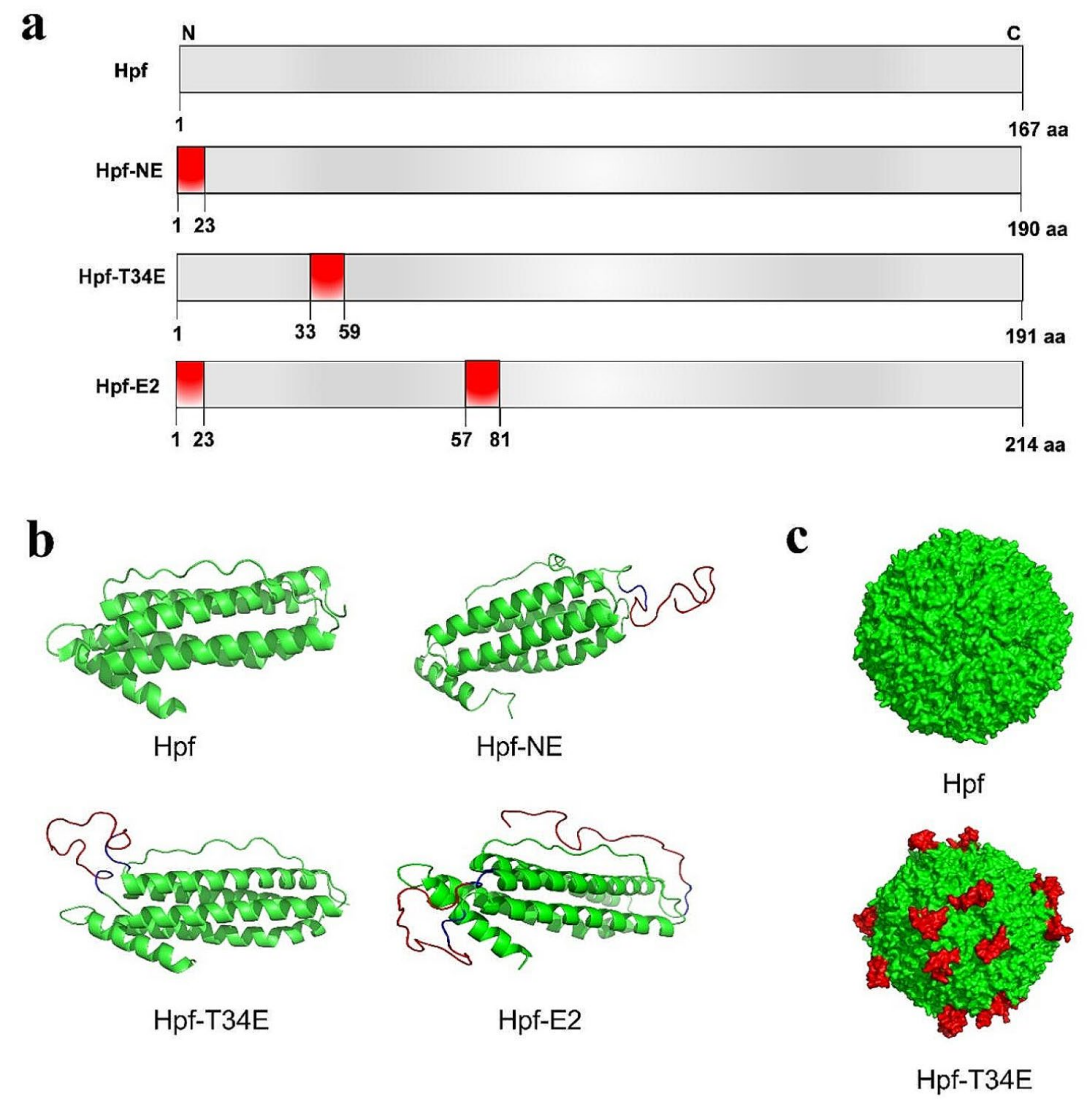
Schematic representation and structural prediction of FNPs. (**a**) Schematic representation showing names (left) and protein lengths in amino acids (right). The insertion positions are marked in red in each Hpf construct. (**b**) The Hpf structure (PDB code: 3bvf) is shown in cartoon representation. Hpf helices αA to αE are coloured in green. The computational models for Hpf constructs are shown in green, except for the inserted peptides and linker (GGS), which are shown in red and blue, respectively. (**c**) 3D models of Hpf and Hpf-T34E. The epitopes and Hpf are represented by red and green, respectively

We postulated that a neutralizing epitope of FMDV can be displayed on the surface of FNPs by rational design of chimeric proteins. To test this hypothesis, we constructed three chimeric protein structural models (Fig. 1a): plasmid pET28a-1, containing the coding sequence of *Helicobacter pylori* J99 ferritin, (GenBank accession code: NP_223316) designated as Hpf; plasmid pET28a-2, carrying coding sequence of the neutralizing epitope peptide GGS**SLPNVRGDLQVLAQKAARP**GGS, inserted at the loop between Hpf helices αA and αB (the construct is designated Hpf-T34E); plasmid pET28a-3, the coding sequence of the neutralizing epitope peptide **SLPNVRGDLQVLAQKAARP**GGS, inserted at the N-terminus of Hpf (designated Hpf-NE); and plasmid pET28a-4, the coding sequence of the neutralizing epitope peptide inserted both at the loop between helices αA and αB and the N-terminus of Hpf (designated Hpf-E2). All recombinant plasmids were sent to a company (BGI, Beijing, China) for synthesis and proofreading. The predicted position and presentation of neutralizing epitopes on the surface of FNPs were obtained by using the I-TASSER suite 5.2 (https://zhanggroup.org/I-TASSER/)[38] (Fig. 1b and c).

### Expression and purification of recombinant proteins

The recombinant expression plasmids were transferred into *E. coli* BL21 (DE3) cells, and expression was induced by 0.5 mM isopropyl β-D-1-thiogalactopyranoside (IPTG) at 16 °C for 12 h. The cells were harvested by centrifugation at 4200 r/min for 10 min and lysed on ice by low-temperature ultrasonic grooving (ATS, Suzhou, China). The precipitate and supernatant were collected by centrifugation at 18,000 r/min for 1 h. Then, the expression of recombinant proteins Hpf, Hpf-NE, Hpf-T34E and Hpf-E2 were analysed by sodium dodecyl sulfate‒polyacrylamide gel electrophoresis (SDS‒PAGE).

The recombinant proteins of Hpf, Hpf-NE and Hpf-T34E were purified by size exclusion chromatography (SEC) (GE, USA) after heat precipitation for 30 min at 65 °C. The purified protein was analysed by SDS–PAGE and Western blotting.

### Transmission electron microscopy

The purified FNPs were diluted in TM buffer (50 mM Tris HCl, pH 7.4, and 10 mM MgCl_2_). For visualization, the FNPs were absorbed on carbon-coated grids, followed by negative staining with 2% aqueous uranyl acetate. The particles were observed at 80 kV under a Hitachi microscope (HT7700, Japan).

### Dynamic light scattering (DLS) analysis of FNPs

Dynamic light scattering experiments were performed on a Malvern Zetasizer Nano ZEN3700 in disposable polystyrene micro-cuvettes (VWRs) using 10 mL of freshly prepared sample solution (0.5 mg/mL, pH = 7.4). After equilibration to 25 °C, three measurements were performed with the instrument. The refractive index (RI) of the dispersant (preset: water) was set to 1.330, and the viscosity (cP) was set to 0.8882. The RI of the particle was set to 1.45. The absorption of the protein was set to 0.001, and both the attenuator and measurement position were controlled by the instrument.

### Western blotting

The purified Hpf, Hpf-NE and Hpf-T34E proteins were separated by 12% SDS‒PAGE and then transferred to a nitrocellulose membrane (ISEQ00010, Merck Millipore), the leftover sites were blocked with 5% skim milk at room temperature for 1 h. After washing the membrane was washed three times with PBST, it was incubated with mouse anti-FMDV VP1 antibodies at 4 °C overnight on a shaker. After washing, the membrane was incubated with an HRP-conjugated goat anti-mouse IgG antibody for 1 h at room temperature. Finally, after three more washes, the membranes were treated with a chemiluminescence (ECL) reagent solution (Thermo Scientific, USA), and the antibody-antigen complexes were exposed and detected with an imaging system (GelDocXR, Bio-Rad, USA).

### Mouse immunization and sample collection

Twenty-four female BALB/c mice, six weeks old (purchased from the Centre of experimental animals of Lanzhou Veterinary Research Institute (LVRI), Chinese Academy of Agricultural Sciences (CAAS)) were divided into 4 groups with six mice in each group: the Hpf, Hpf-T34E, Hpf-NE and PBS groups. The mice were subcutaneously immunized on the back with 50 µg of antigen emulsified with ISA 206 adjuvant (Seppic, Paris, France) on day 0, and the immunization was boosted on day 14. In the control group, the mice were immunized with an equal volume of sterilized phosphate buffered saline (PBS) after emulsification with adjuvant. Blood samples were collected through the tail vein at 0, 7, 14, 21, 28, 35 and 42 (day) post vaccination (dpv). The mice were sacrificed on day 42 to isolate splenocytes (Fig. 3a).

### Immunization, challenge and sample collection in guinea pigs

The immune response and protection conferred by the Hpf-T34E protein were assessed in guinea pigs weighing 200–250 g. Fifteen guinea pigs (purchased from the Centre of experimental animals of Lanzhou Veterinary Research Institute (LVRI), Chinese Academy of Agricultural Sciences (CAAS)) were randomly divided into three groups with five guinea pigs in each group: the Hpf-T34E, IV and PBS groups. The Hpf-T34E group was immunized with Hpf-T34E protein (100 µg) emulsified with ISA 206 adjuvant (Seppic, Paris, France). The IV group was immunized with a commercial inactivated FMD vaccine (IV) (China Agricultural Vet. Bio. Science and Technology Co., Ltd, China) as a positive control group. The PBS group was immunized with PBS mixed with ISA 206 adjuvant as a negative control group. The guinea pigs were immunized intramuscularly in the thigh on day 0, and the immunization was boosted on day 21. Blood samples were collected at 0, 21, 35 and 42 dpv.

Guinea pigs were housed in separate units of the high-containment facility, and were subcutaneously and intradermally challenged with 0.2 ml 100 GPID_50_ (guineapig infective dose 50) of FMDV isolate O/Mya98/BY/2010 (GenBank accession code: JN998085.1) on the left sole of the back at the third week after boost vaccination. All the guinea pigs were kept in isolated hutches and examined for 7 days. The guinea pigs were sacrificed at 49 dpv to isolate the heart and spleen. Lesions on the left back soles were considered indicators of partial infection, and those on both back soles were considered indicators of whole-body infection.

### Detection of specific antibodies by ELISA

The detection of serum antibodies against FMDV was performed by enzyme-linked immunosorbent assay (ELISA). The inactivated FMDV (purified 146 S particles) was coated with coating buffer (Solarbio Life Science, Beijing, China) and incubated overnight at 4 °C. After being blocked with 5% skim milk for 1 h at 37 °C, the plate was washed three times with PBS–0.1% Tween 20 (PBST) and incubated with serial twofold dilutions of each serum sample prepared in PBS, for 1 h at 37 °C. After six washes with PBST, the plates were incubated at 37 °C for 45 min with HRP-conjugated goat anti-mouse IgG (ImmunoWay, USA) for mouse sera, and HRP-conjugated rabbit anti-guinea pig IgG (ImmunoWay, USA) for guinea pig sera, both at a 1:5000 dilution in PBST. After another wash, 100 µL of 3, 39, 5, 59-tetramethylbenzidine was added to each well as a chromogenic substrate solution. The reaction was stopped with 50 µL of 0.5 M H_2_SO_4_ after incubation at room temperature for 30 min. The optical density at 450 nm (OD value) of each well was measured using an ELISA reader.

### Virus neutralization test (VNT)

Briefly, each serum sample was used in a monolayer of BHK-21 cells to perform the neutralization test. One hundred microlitres of the heat inactivated was serially diluted twofold from 1:4 − 1:1024. Then, the diluted serum was mixed with an equal volume of 100 TCID_50_ of FMDV and incubated for 1 h at 37 °C. Subsequently, 100 µL of the mixture was transferred to BHK-21 cells in a 96-well plate and incubated for 1 h at 37 °C. The cell supernatant was discarded, and after three washes, DMEM containing 2% FBS was added. Each serum sample was analysed in triplicate. After incubation for 48–72 h, the endpoint titres were calculated as the reciprocal of the last serum dilution to neutralize 100 TCID_50_ of homologous FMDV in 50% of the wells.

### T-Lymphocyte proliferation assay

A T-lymphocyte proliferation assay was performed with a CCK-8 (Cell Counting Kit-8) assay. The spleens were removed in a sterile manner and ground through a sterile cuprous 200-mesh. The mixture of splenocytes was immersed in RPMI 1640 medium supplemented with 10% FBS, homogenized and centrifuged at 400 × *g* for 10 min. The pellets were discarded, and the buoyant cells were washed three times in RPMI 1640 medium supplemented with 10% FBS. After red blood cell lysis, 3 × 10^5^ splenocytes were distributed in triplicate wells of 96-well flat-bottomed plates. Cells were stimulated with either purified FMDV-VP1 protein (10 µg/well) or purified Hpf (10 µg/well). Triplicate wells with 3 × 10^5^ cells without protein were used to estimate nonspecific activation. As a positive control, triplicate wells with 3 × 10^5^ cells were stimulated with 50 µL of ConA (2.5 µg/well). The plate was incubated at 37 °C, and 5% CO_2_ for 72 h, followed by incubation with CCK-8 for 4 h, and the absorbance was determined at 450 nm. The stimulation indices (SIs) were calculated using the following formula: SI = (OD _sample well_ – OD _blank well_) / (OD _negative well_ – OD _blank well_) at OD_450 nm_ with three technical repeats.

### In vitro cytokine release assay

The lymphocytes prepared in the previous step were seeded into 24-well plates at 1 × 10^6^ cells/well. Cytokine secretion was stimulated with 10 µg/mL purified FMDV VP1 protein. After the plate was incubated at 37 °C and 5% CO_2_ for 72 h, the concentrations of IFN-γ, IL-2, IL-4 and TNF-α in the supernatants were determined using a commercial ELISA kit (DAKEWE, China). The data calculations were performed according to the manufacturers’ instructions.

### Quantitative real-time PCR (qPCR)

RNA was extracted from the samples using TRIzol reagent (Invitrogen, USA). Quantitative real-time PCR was performed using a One Step PrimeScript RT‒PCR kit (Takara, China), according to the manufacturer’s protocol. qPCR amplification was performed using a CFX96 Touch RT‒PCR Detection System (Bio-Rad, USA). The reaction mixtures contained RNA (10–100 ng), a TaqMan probe (FAM-TCCTTTGCACGCCGTGGGAC-TAMRA) (Sangon Biotech, China), sense primers (ACTGGGTTTTACAAACCTGTGA) and reverse primers (GCGAGTCCTGCCACGGA) (20 µmol/L) (Sangon Biotech), and RNase-free water to a total volume of 20 µl. The PCR cycling conditions were as follows: 42 °C for 5 min; 95 °C for 10 s; and 40 cycles of 5 s at 95 °C, 20 s at 60 °C and 30 s at 72 °C. The data represent the results from one representative triplicate experiment.

### Statistical analysis

The statistical analysis was performed using column analysis t tests and two-way analysis in GraphPad Prism 8.0 (GraphPad Software, Inc., USA). All the data are presented as the means ± standard deviations (SDs). Various degrees of significance were designated as follows: * *p* < 0.05, ** *p* < 0.01, *** *p* < 0.001 and ns indicates no significant difference.

## Results

### Design and preparation of the FNPs

**Fig. 2:**
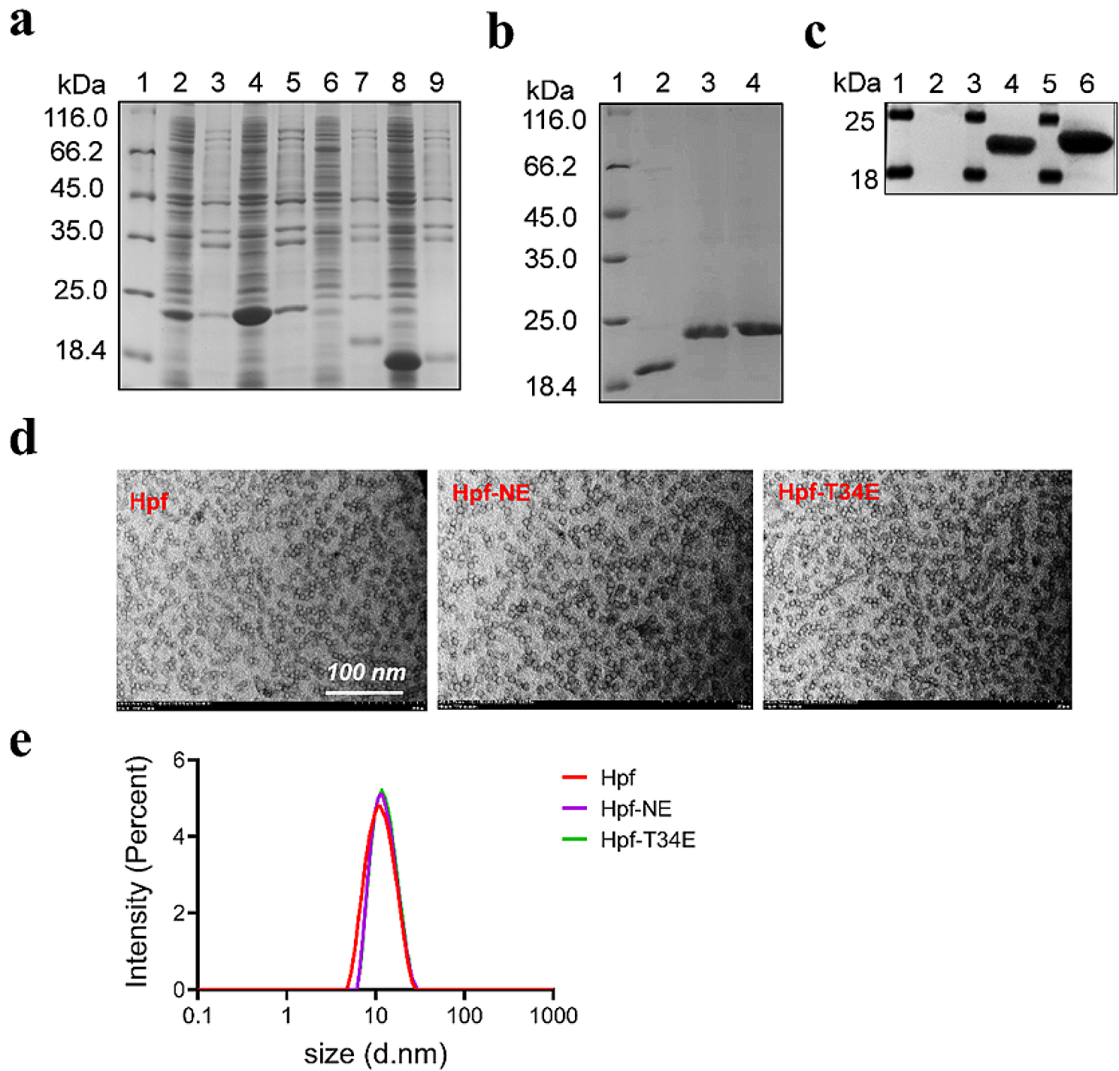
Preparation and characterization of FNPs. (**a**) SDS‒PAGE analysis of the solubility of four recombinant proteins. Lane 1, protein ladder; lanes 2–3, expression of Hpf-NE in the supernatant and precipitate of cell lysates, respectively; lanes 4–5, expression of Hpf-T34E in the supernatant and precipitate of cell lysates, respectively; lanes 6–7, Hpf-E2 expressed in the supernatant and precipitate of cell lysates, respectively. lanes 8–9, Hpf expressed in the supernatant and precipitate of cell lysates, respectively. (**b**) SDS‒PAGE analysis of the purity of the Hpf, Hpf-NE and Hpf-T34E proteins. Lane 1, protein ladder; Lane 2, purified Hpf; Lane 3, purified Hpf-NE; Lane 4, purified Hpf-T34E. (**c**) Antigenicity was verified using an anti-FMDV-VP1 rabbit polyclonal antibody. Lanes 1, 3, and 5, protein ladder; Lane 2, purified Hpf; Lane 4, purified Hpf-NE; Lane 6, purified Hpf-T34E. (**d**) Electron microscopy analysis of FNPs. Negatively stained purified proteins corresponding to Hpf, Hpf-NE and Hpf-T34E; scale bar = 100 nm. (**e**) Dynamic light scattering (DLS) results of FNPs.

We generated recombinant prokaryotic expression plasmids for different FNP constructs (Fig. 1a). The foreign amino acid sequence was incorporated into the Hpf peptide PLPNVRGDLQVLAQKAARP (19 aa), which contains the FMDV neutralizing epitope from serotype O (residues 140–158 from the VP1 capsid protein). The inserted immunogenic epitopes were flanked by the amino acids glycine and serine (GGS) as a flexible linker intended to facilitate ferritin assembly. Based on this report [14], foreign epitopes could be inserted into the N-terminus, or the loop between Hpf helices αA and αB lying on the surface of the nanoparticle [14]. The generation of three chimeric mutants was achieved by the insertion of either a single neutralizing epitope or both, targeting specific regions within the ferritin protein sequence. These regions include the N-terminal end (Hpf-NE), located between amino acids Thr 33 and His 34 in the loop between Hpf helices αA and αB (Hpf-T34E), as well as both the N-terminal end and preceding His 34 (Hpf-E2). The structure of the Hpf, Hpf-NE, Hpf-T34E and Hpf-E2 proteins are predicted (Fig. 1b), and the presence of neutralizing epitopes on the surface of the Hpf-T34E nanoparticles was determined by using the I-TASSER suite 5.2 (Fig. 1c). SDS‒PAGE and Western blotting were performed to evaluate the solubility and immunogenicity of the recombinant proteins. The SDS‒PAGE results indicated that the recombinant proteins obtained were of the expected molecular weights for Hpf (18 kDa) (Fig. 2a, lanes 8–9), Hpf-T34E (22 kDa) (Fig. 2a, lanes 4–5) and Hpf-NE (22 kDa) (Fig. 2a, lanes 2–3). These three recombinant proteins were expressed in soluble form and purified by size exclusion chromatography (SEC) (Fig. 2b). Hpf-E2 (24 kDa) (Fig. 2a, lanes 6–7) was not expressed. Additionally, the Hpf-T34E and Hpf-NE proteins could be recognized by anti-FMDV VP1 antibodies (Fig. 2c). Electron microscopic analysis also revealed that the Hpf-T34E and Hpf-NE constructs self-assembled into nanoparticles with a diameter of approximately 15 nm, exhibiting morphological similarities to Hpf (Fig. 2d), which was consistent with the results of DLS (Fig. 2e).

### Evaluation of the immunogenicity of the FNPs in mice

**Fig. 3:**
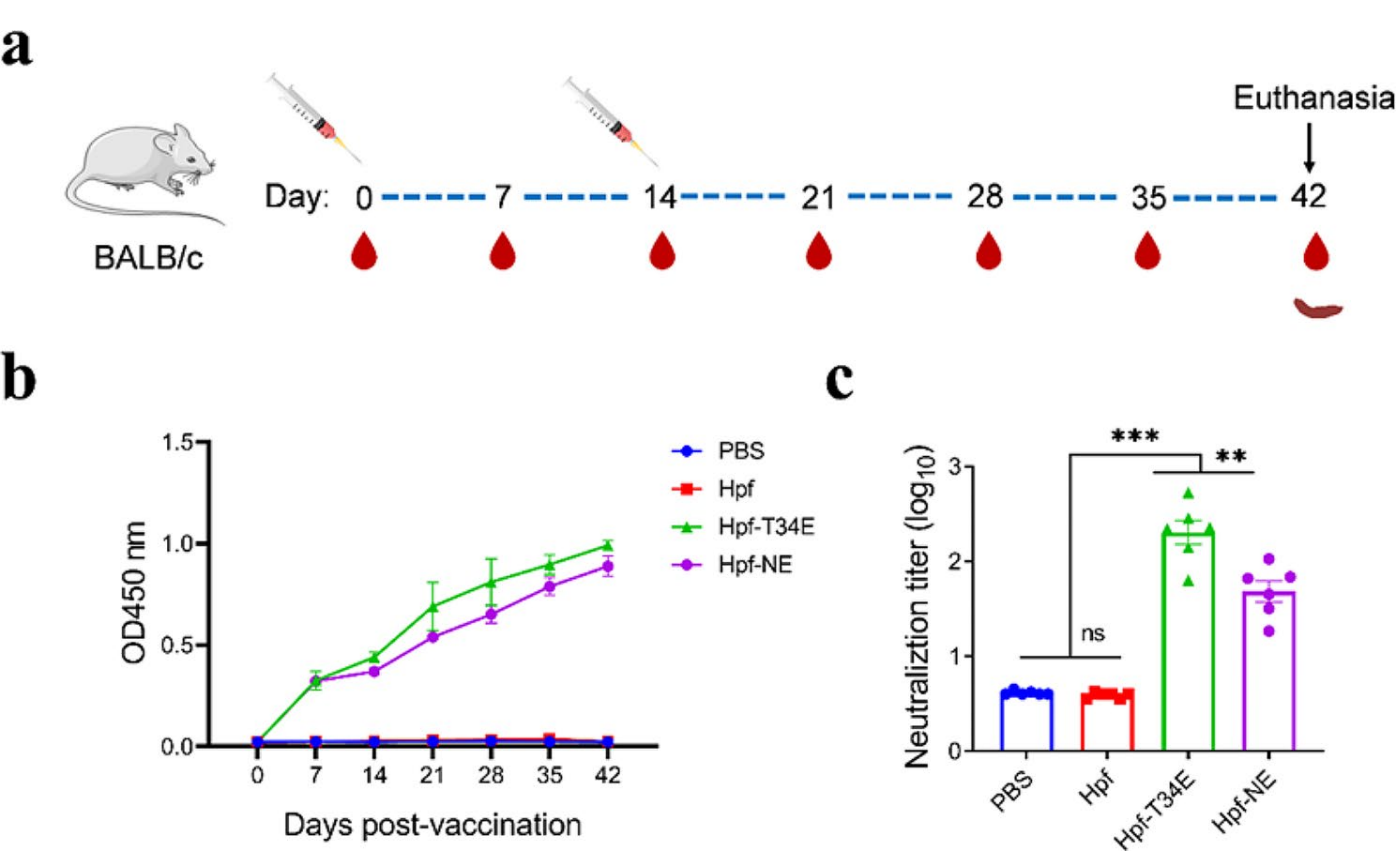
The IgG and neutralizing antibody levels in the serum of mice immunized with FNPs. (**a**) Diagram of the immunization program and sampling. (**b**) The levels of IgG in mouse serum were measured by iELISA. (**c**) The titres of neutralizing antibodies in mouse serum were detected via virus neutralization tests. * *p* < 0.05; ** *p* < 0.01; *** *p* < 0.001, and ns indicates no significant difference

To evaluate the specific immunogenicity of the generated FNP vaccine candidates, specific pathogen-free mice were selected and divided into 4 groups: Hpf, Hpf-T34E, Hpf-NE and PBS groups. Two vaccinations were administered at 0 and 14 days (Fig. 3a). Serum samples were collected at 0, 7, 14, 21, 28, 35 and 42 dpv, and the IgG antibodies were detected by ELISA. The results showed that the titres of IgG antibodies increased gradually in the Hpf-NE and Hpf-T34E groups after vaccination, and the level of IgG in the Hpf-T34E group was slightly greater than that in the Hpf-NE group. All preimmunized serum samples, as well as the sera from the PBS group and the Hpf group were negative for antibodies (Fig. 3b).

Concerning the induction of FMDV neutralizing antibodies, serum samples were collected at 42 dpv, and the titres of neutralizing antibodies were detected by a virus neutralization test (VNT). The results showed that the titres of neutralizing antibodies in the Hpf-T34E group were greater than those in the Hpf-NE group (*p* < 0.01). Indeed, this was the only Hpf-T34E group that exhibited average VNT titres > 2 log_10_. No detectable neutralizing activity was detected in the PBS group or the Hpf group (Fig. 3c).

**Fig. 4:**
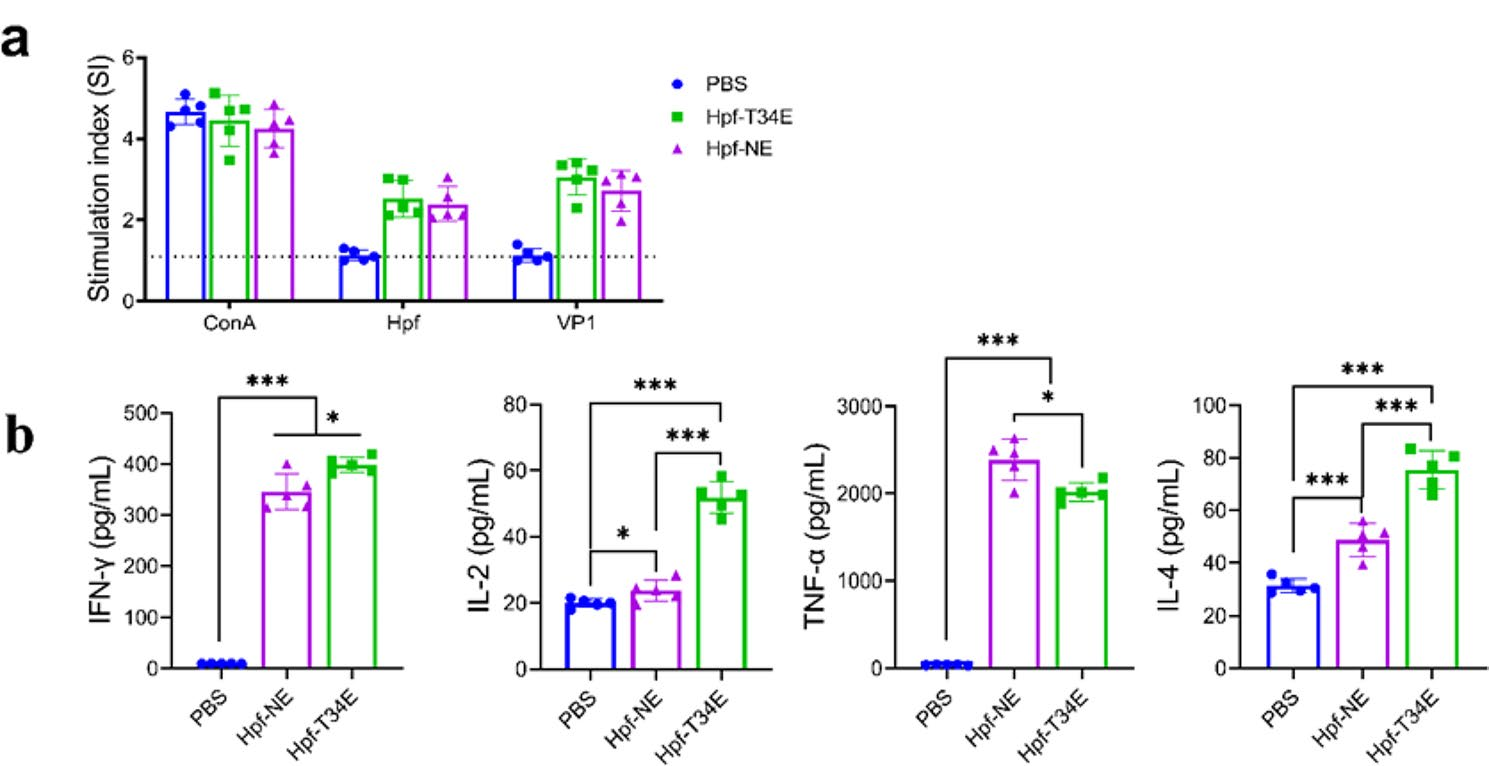
Detection of lymphocyte proliferation and cytokine levels in FNP-immunized mice. (**a**) The results of the lymphocyte proliferation assay on day 28 dpv; the dashed line indicates the threshold above which the lymphoproliferative response was considered positive. (**b**) Detection of the levels of mouse cytokines (INF-γ, IL-2, IL-4, and TNF-a). * *p* < 0.05; ** *p* < 0.01; *** *p* < 0.001, and ns indicates no significant difference

Lymphocytes were collected from the Hpf-NE group, Hpf-T34E group and PBS group at 42 dpv. Lymphoproliferation assays were completed in vitro with the FMDV VP1 protein and Hpf protein. Lymphocytes from the Hpf-T34E group and Hpf-NE group elicited a highly specific response (SI > 2) to the Hpf protein. Similarly, a considerable level of specific proliferative response was found after in vitro stimulation with the FMDV VP1 protein (SI > 2.5) (Fig. 4a). Taken together, these results indicated that mice were vaccinated with the Hpf-NE protein and that the Hpf-T34E protein could induce a detectable specific T-cell response.

The ability of lymphocytes to secrete cytokines after stimulation is positively correlated with their functions. The levels of interferon gamma (IFN-γ), interleukin 2 (IL-2), interleukin 4 (IL-4) and TNF-α following the stimulation of splenocytes with FMDV VP1 protein in vitro were determined using ELISA kits from the Hpf-NE, Hpf-T34E and PBS groups at 42 dpv. The concentrations of IFN-γ, IL-2, IL-4 and TNF-α in the Hpf-NE group and Hpf-T34E group were greater than those in the PBS group(*p* < 0.001). A high titre (300–400 pg/ml) of IFN-γ was detected in the Hpf-T34E group and Hpf-NE group, and the concentration of IFN-γ in the Hpf-T34E group was greater than that in the Hpf-NE group (*p* < 0.05). Similar to the results for IFN-γ, the concentrations of IL-2 and IL-4 were significantly greater in the Hpf-T34E group. In contrast, the concentration of TNF-α in the Hpf-T34E group was lower than that in the Hpf-NE group (*p* < 0.05) (Fig. 4b).

### Evaluation of the immunogenicity of the Hpf-T34E protein in guinea pigs

**Fig. 5:**
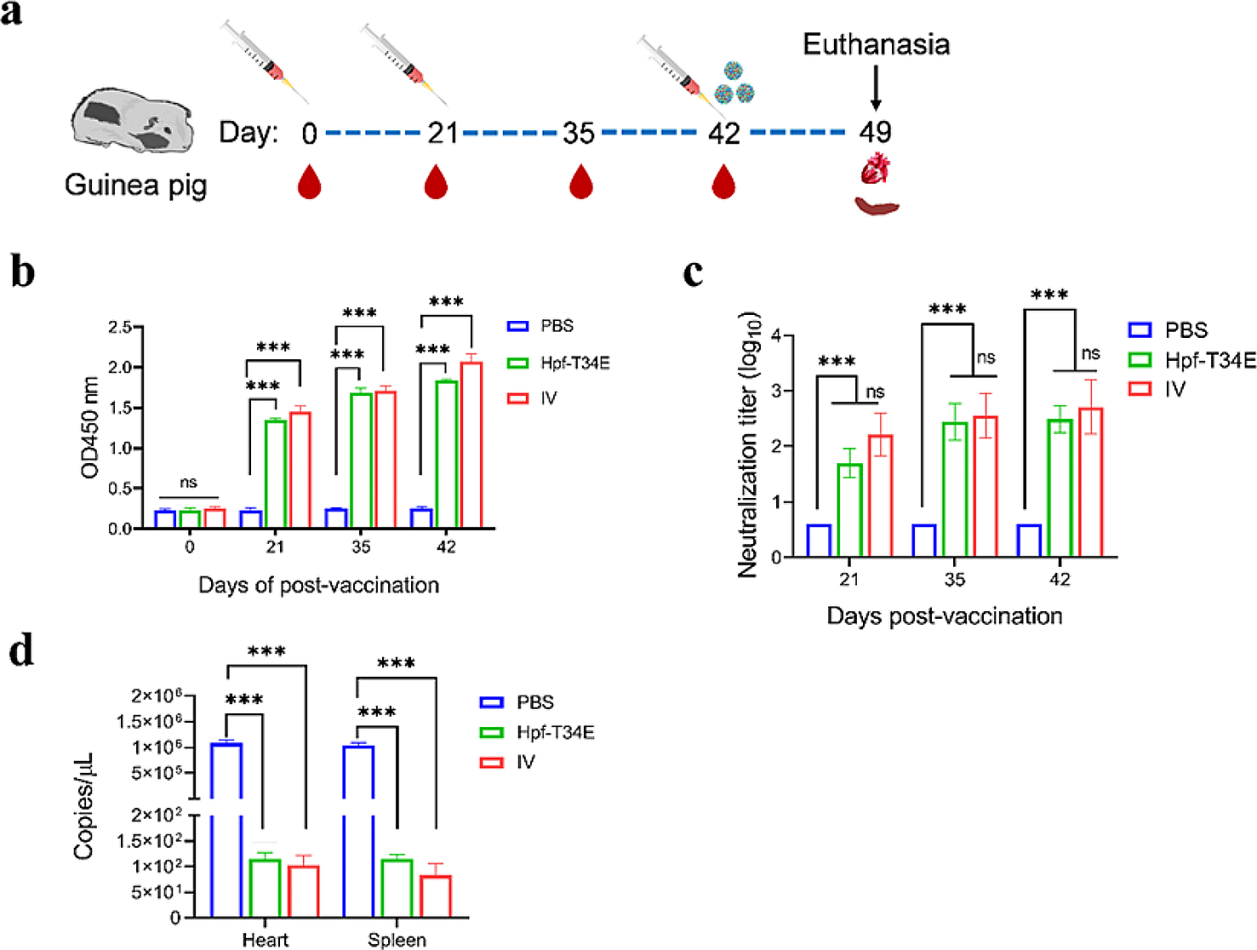
Evaluation of the immunity of Hpf-T34E in guinea pigs. (**a**) Experimental schema of immunization, sampling, and challenge. (**b**) The levels of IgG in guinea pig serum were measured by ELISA. (**c**) The titre of neutralizing antibody in guinea pig serum was detected via virus neutralization tests. * *p* < 0.05; ** *p* < 0.01; *** *p* < 0.001, and ns indicates no significant difference. (**d**) Detection of the viral load in guinea pigs after challenge. The data are presented as the means ± SEs. Significance was determined by ANOVA (*** *P* < 0.001)

Based on the results obtained from the mice, the Hpf-T34E protein was selected for further evaluation in guinea pigs, which is one of the most relevant FMDV animal models. Specific pathogen-free guinea pigs were selected and divided into 3 groups: the Hpf-T34E, IV and PBS groups. Two vaccinations were administered at 0 and 21 days. Subsequently, the guinea pigs were challenged on day 42 (Fig. 5a). Two additional groups (IV and PBS) were included in the experiment as FMDV infection controls. Serum samples were collected at 0, 21, 35 and 42 dpv, and the IgG antibodies were detected by ELISA. The results showed that the IgG antibody titers in the IV group and Hpf-T34E group were significantly greater than those in the PBS group (*p* < 0.001), but there was no significant difference in the antibody levels between the IV group and Hpf-T34E group at 35 dpv (Fig. 5b).

The titre of neutralizing antibodies was detected in guinea pig serum at 21, 35, and 42 dpv (Fig. 5c). The results showed that neutralizing antibodies were elicited in the Hpf-T34E group and IV group at 21 dpv. There was no neutralizing antibody produced in the PBS group. After the boost, the levels of neutralizing antibodies were significantly increased in the Hpf-T34E group and IV group at 35 dpv. However, the levels of neutralizing antibodies were not significantly different between the IV group and the Hpf-T34E group at 42 dpv.

### Protection against FMDV challenge in guinea pigs

Protection efficacy was measured by carefully observing and recording clinical signs in guinea pigs for 7 days after challenge. Guinea pigs were considered partially protected when lesions only appeared on the sole of the left backfoot, and were considered wholly protected when lesions were not observed on either sole of the backfoot. As expected, the guinea pigs in the PBS group showed full FMD signs (the lesion appeared on both back soles) upon challenge. One guinea pig in the Hpf-T34E group exhibited lesions on the sole of left backfoot in the Hpf-T34E group. However, the guinea pigs in the IV group were fully protected (Table 1).

In the IV group, the number of virus copies in the heart and spleen was lowest compared with all the other groups. The number of virus copies in the Hpf-T34E and IV groups was significantly lower than that in the PBS group at 7 dpi (*p* < 0.001) (Fig. 5d). Overall, the results obtained indicated that vaccination with the Hpf-T34E protein had a protection rate of 80% (4/5) in guinea pigs.

**Table 1:** Protection of Guinea pigs after challenge with FMDV

Number of guinea pigs	PBS	Hpf-T34E^a^	IV^b^
FMD signs			
1	Both back soles	None	None
2	Both back soles	None	None
3	Both back soles	None	None
4	Both back soles	Sole of left backfoot	None
5	Both back soles	None	None
Protective degree			
1	NO	Full	Full
2	NO	Full	Full
3	NO	Full	Full
4	NO	Partial	Full
5	NO	Full	Full
Rate of protection(%)	0(0/5)	80%(4/5)	100%(5/5)

a: purified recombinant protein Hpf-T34E

b: commercial inactivated FMD vaccine (IV) (China Agricultural Vet. Bio. Science and Technology Co., Ltd, China)

## Discussion

FMD is an important animal infectious disease, that threatens livestock production worldwide [39]. Currently commercially available inactivated vaccines have various limitations, such as the potential risk associated with the production and distribution of vaccines, and the difficulty of quickly preparing a vaccine against the new emerging epidemic FMDV strain [5] This has greatly limited and hindered the prevention, control and eradication of FMD. An innovative approach to produce safe and effective vaccines to prevent the occurrence and spread of FMDV is urgently needed. NPs provide an improved vaccine platform for the delivery of antigens [40] and have been developed clinically to combat several infectious diseases [11–13, 24, 32]. The naturally self-assembling FNPs composed of 24 identical polypeptides in well-ordered arrays have been highly utilized in novel genetically engineered subunit vaccines and in inducing a broadly neutralizing antibody [11–13].

In the present study, we evaluated the ability of engineered FNPs displaying a neutralizing epitope to elicit an immune response and protection against FMDV. To achieve this goal, one well-defined immunogenic epitope, the immunodominant epitope located within the G-H loop of the FMDV VP1 capsid protein, was used. This epitope has been showed to induce cross-reactive specific antibodies against a set of O-type FMDV topotypes [36, 41–43]. Thus, it is important to explore whether FNPs could serve as effective vaccine vectors to deliver the dominant FMDV epitope-inducing protection. In this study, the recombinant proteins Hpf, Hpf-NE and Hpf-T34E were efficiently expressed and readily assembled into NPs (Fig. 2d). This result further confirms and extends the reported versatility of FNPs, which can accept foreign sequences at two different insertion sites (the N-terminus and between helices αA and αB) [14]. However, our results also showed that simultaneous insertion of foreign sequences at two FNP sites affected protein expression (Fig. 2a). In addition, we found that the expression level of the Hpf-T34E protein was obviously greater than that of the Hpf-NE protein. This result suggests that the specific site of exogenous peptide incorporation within Hpf may influence protein expression levels.

The results of the humoral immunoassay showed that both mice (Fig. 3b) and guinea pigs (Fig. 5b) vaccinated with engineered FNPs elicited effective FMDV-specific antibody responses. The Hpf-NE protein and Hpf-T34E protein exhibited similar FMDV-specific antibody titres in mice (Fig. 3b), suggesting that both surface-exposed insertion sites of Hpf can induce antibody responses. Furthermore, the Hpf-NE protein and Hpf-T34E protein also induced high-titers of neutralizing antibodies in mice (Fig. 3c). This neutralizing immune response was significantly greater than that previously reported [16], who inserted the FMDV G-H loop (aa 141–160) at the N-terminus of Hpf. This result may be because the neutralizing epitope of Hpf-T34E was inserted into Hpf helices αA and αB, which are located in a loop region. This design maintains the correct conformation of the G-H loop and facilitates the triggering of the neutralizing activity of the epitope. Although it is generally accepted that protective immunity to FMDV is mostly associated with the neutralizing antibody response, cellular immunity also plays an important basic regulatory role in inducing antibody production and persistence [44]. The Hpf-T34E protein induced a significant lymphocyte proliferative response and robust levels of IFN-γ, IL-2, IL-4 and TNF-α (Fig. 4). This result further confirmed the ability of FNPs to evoke a T-cell response. In addition, the results suggested that different insertion positions of epitope peptides in FNPs may have different effects on cytokine induction. Furthermore, the Hpf-T34E protein also elicited a robust neutralizing antibody response in guinea pigs (Fig. 5c). Clinical evaluation of the animals after virus challenge was performed according to a previous report on FMDV-challenged guinea pigs [45]. Guinea pigs immunized with Hpf-T34E exhibited an 80% protection rate (Table 1). In fact, the induction of anti-FMDV neutralizing antibodies triggered by such FNPs in natural hosts remains to be evaluated, as these antibodies have only been detected in murine and guinea pig models.

## Conclusion

In this study, our findings suggested that the use of FNPs containing a neutralizing epitope represents a promising strategy for eliciting a protective immune response against homologous FMDV challenge. Furthermore, inserting the epitope between the αA and αB helices of Hpf could enhance its expression, maintain its conformational stability and promote stronger immune responses. Based on these encouraging results, we optimistically anticipate that FNPs containing the dominant epitope of FMDV may emerge as safe and effective FMD vaccines in the future.

## Data Availability

Data will be made available on request.

## Abbreviations

FMD: Foot-and-mouth disease
FMDV: Foot-and-mouth disease virus
Hpf: *Helicobacter pylori ferritin*
FNPs: Ferritin-based nanoparticles
BHK-21: Baby hamster kidney-21
aa: Amino acids
SEC: Size exclusion chromatography
VNT: Virus neutralization test
DMEM: Dulbecco’s modified Eagle’s medium
dpv: Day post vaccination
